# The Effectiveness of Repetition or Multiplicity of Different Surgical and Non-Surgical Procedures Compared to a Single Procedure Application in Accelerating Orthodontic Tooth Movement: A Systematic Review and Meta-Analysis

**DOI:** 10.7759/cureus.23105

**Published:** 2022-03-12

**Authors:** Doa'a Tahseen Alfailany, Mohammad Y Hajeer, Ossama Aljabban, Luai Mahaini

**Affiliations:** 1 Department of Orthodontics, University of Damascus Faculty of Dentistry, Damascus, SYR

**Keywords:** low-level laser therapy, mops, multiple osteoperforation, corticotomy, non-surgical acceleration, surgical acceleration, repeated application, combined application, acceleration, arthodontic tooth moevement

## Abstract

In this study, we aimed to assess the current scientific evidence concerning the effectiveness of combining two acceleration techniques or repeating an acceleration procedure in comparison with the single application in terms of the speed of the orthodontic tooth movement (OTM).

We performed a comprehensive electronic search to retrieve relevant studies on 10 databases. Randomized controlled trials (RCTs) on fixed orthodontic treatment patients who received multiple types of acceleration techniques or underwent a repeated acceleration procedure compared to a single application were included. Version 2 of the Cochrane risk-of-bias tool for randomized trials (RoB 2) was used for assessing the risk of bias of retrieved studies.

A total of six RCTs were included in this review. Regarding multiple acceleration methods, it seems that the combination of low-level laser therapy (LLLT) with a surgical technique outperforms the single application of each technique separately. Additionally, the combination of two surgical interventions may have a synergistic effect leading to reduced treatment time compared to the application of a single intervention. Regarding acceleration method repetition, it seems that the re-application of surgical procedures twice is more efficient than the single application. The meta-analysis showed a non-significant difference in the canine retraction rate between the four-weekly micro-osteoperforations (MOPs) (three times of applications) and both the eight-weekly MOPs (two times of applications) [mean difference (MD) = 0.24; 95% CI: -0.2-0.77; p = 0.36], as well as 12-weekly MOPs (two times of applications) (MD = 0.06; 95% CI: -0.14-0.27; p = 0.55).

Based on very low evidence, combining two acceleration techniques is superior over a single application in accelerating tooth movement. Again, very low evidence suggests that the efficacy of repetition of surgical procedures twice and three times is similar. Further high-quality RCTs are required to assess the benefit of repeating an acceleration procedure or combining two different methods. In addition, more insight is needed into the possible side effects associated with the repetition or multiplicity of procedures.

## Introduction and background

Comprehensive orthodontic treatment for moderate to severe cases of malocclusion usually lasts for more than 18 months, taking into account several factors that lead to a significant difference [1]. Elongating orthodontic treatment may lead to white spots formation, dental caries, apical root resorption, periodontal disease, pain, and discomfort [2,3]. Moreover, many adult patients wish to finish orthodontic treatment faster for aesthetic or social reasons [4]. Hence, many attempts have been made to shorten orthodontic duration: local or systemic administration of pharmacological substances, mechanical or physical stimulation, and surgical interventions [5].

Pharmacological interventions depend on the injection of local substances, which is related to the biological response occurring during tooth movement. The efficacy of pharmacological molecules has been investigated in many human and animal studies [6]. Many of these substances have proven their effectiveness, e.g., prostaglandins, which are inflammatory mediators that increase the number of osteoclasts leading to stimulating bone resorption and acceleration of the orthodontic tooth movement (OTM). Prostaglandin injection has been proven to be associated with pain and dose-dependent root resorption [7]. Also, pharmacological molecules that have proven effective in accelerating OTM include vitamin D [8] and hormones such as the parathyroid hormone (PTH) [9]. On the other hand, relaxin, which is considered to be a hormone, has many roles as it helps during childbirth as well as impacts some physiologic functions such as collagen turnover, angiogenesis, and antifibrosis, in addition to its role in soft tissue remodeling [10]. It affects the OTM by increasing the collagen in the tension site and decreasing it in the compression site. The mechanism of relaxin's effect on acceleration OTM remains unclear. However, one of the studies that investigated the effect of relaxin in humans showed that its injection was not effective in accelerating OTM [10].

The physical methods rely on using device-assisted therapy and include direct electric currents, pulsed electromagnetic field, vibration [11], and low-level laser, which has been widely investigated in many research projects [12] and has proven to be effective in its stimulating efficacy in alveolar bone resorption and formation processes by increasing osteoclast and osteoblast numbers, leading to the acceleration of OTM [13].

The surgical procedures are considered the most clinically effective methods, and they have been rigorously tested several times in terms of the possibility of significantly decreasing treatment duration [3]. However, the surgical intervention includes various procedures such as conventional corticotomy [5,14], dentoalveolar distraction [15], periodontal distraction [16], interseptal alveolar surgery [17], accelerated osteogenic orthodontics [18], piezocision [2,19], corticision [20], and micro-osteoperforations (MOPs) [21]. All these procedures depend on the same principle, "regional acceleratory phenomenon" (RAP), which was first described by Forest and is based on the principle that the occurrence of surgical injury to the alveolar bone may temporarily speed up OTM [22]. The RAP was described as a transient stage of localized soft and hard tissue remodeling that resulted in the rebuilding of the injured sites to a normal state through recruitment of osteoclasts and osteoblasts by way of local intercellular mediator mechanisms involving precursors [22]. This mechanism does not involve any secondary healing by fibrous tissue formation.

The focus of several recently published systematic reviews (SRs) has been on evaluating the effectiveness of different individual interventions for orthodontic acceleration, both surgical [23-25] and non-surgical [26,27]. Mohaghegh et al. [28], in their recent SR, discussed the effect of single and multiple MOPs on the rate of OTM. However, this SR was oriented only toward the MOPs procedure although repetition can be performed for other acceleration procedures such as traditional corticotomy, flapless corticision, flapless piezocision corticotomy, and high-intensity laser therapy-assisted corticotomy. In addition, no SR has evaluated the effect of combining different procedures, such as combining surgery with low-level laser therapy (LLLT) or surgery with vibrational devices. Hence, we performed this SR to address the following focused review question: Does the repetition of an acceleration procedure or the combination of different procedures outweigh a single application in patients undergoing fixed orthodontic treatment? In light of this objective, we aimed to critically appraise the available evidence regarding the effectiveness of multiplicity or repetition of acceleration procedures in comparison with the single application for speeding up orthodontic treatment.

## Review

Materials and methods

Initially, a PubMed® scoping search was carried out to verify the existence of similar SRs and to check out potentially eligible trials before writing the final SR protocol. The search results indicated the presence of one potentially eligible study and the absence of any similar SRs about the same topic. Registration of this review with PROSPERO was performed (https://www.crd.york.ac.uk/prospero/display_record.php?ID = CRD42021274314; 2021: CRD42021274314). This SR was prepared in accordance with the Cochrane Handbook for Systematic Reviews of Interventions [29], and the Preferred Reporting Items for Systematic Reviews and Meta-Analyses (PRISMA) guidelines [30,31].

Eligibility criteria

Criteria of Exclusion and Inclusion Were Applied According to the PICOS (Patient/Population, Intervention, Comparison, and Outcomes) Framework as Follows:

Participants: healthy patients of both genders (regardless of age, malocclusion type, and racial group) undergoing fixed orthodontic treatment (either extraction- or non-extraction-based treatments).

Type of interventions: first theme: multiple methods of acceleration [two or more different methods of acceleration even they fell within the same category of acceleration (e.g., corticision followed by MOPs, both being surgical interventions)]. Second theme: repeated acceleration using a single method of acceleration at different time intervals (in the interventional group) compared to a single application of this method (or less frequent applications) in the control group.

Comparisons: first theme: only a single method of acceleration from any category. Second theme: only one application of the acceleration procedure without repetition (or with less frequent applications compared to what was applied in the control group).

Outcomes: primary outcomes were the rate of tooth movement (RTM), the time of tooth movement (TTM), or any equivalent measurement. Secondary outcomes: a complication reported by patients (e.g., pain, discomfort, and other related experiences), or gingival and periodontal problems including periodontal index (PI), gingival index (GI), attachment loss (AT), gingival recession (GR), and periodontal depth (PD), or undesired tooth movement (tipping, torquing, rotation), or anchorage loss, or bone/root changes including bone density (BD), bone resorption (BR), root resorption (RR), or long-term treatment stability.

Study design: we took into account all randomized controlled trials (RCTs) without any restrictions regarding the publication year or the language used.

Exclusion criteria: retrospective studies, non‑English language trials, in vitro studies, animal studies, reviews and technique description papers, editorials, personal opinions, case reports or case series reports, and finite element analysis articles were excluded.

Search strategy

An electronic search of databases [The Cochrane Central Register of Controlled Trials (CENTRAL), EMBASE®, Scopus®, PubMed®, Web of Science™, Google™ Scholar, Trip, OpenGrey (to determine the grey literature), and PQDT OPEN from pro-Quest® (to determine dissertations and theses)] was carried out in August 2021 in the English language only with no time limitation. Scrutiny of selected trials reference lists' was done to investigate if any scientific paper was inadvertently missed during electronic research. Also, manual searching was conducted in the same period; the American Journal of Orthodontics and Dentofacial Orthopedics, the European Journal of Orthodontics, and the Angle Orthodontist. ClinicalTrials.gov and World Health Organization International Clinical Trials Registry Platform Search Portal (ICTRP) were also screened electronically to recover any unpublished studies or recently completed research work. More details about the electronic search strategy are provided in Table 1.

Study selection and data extraction

Two reviewers (DTA and MYH) separately evaluated the studies' eligibility, and in instances of disagreement, a third author (OJ) helped in resolving this. At first, only titles and abstracts were checked. Subsequently, the full text of trials that appeared to be pertinent was evaluated and selected for inclusion, as well as titles or abstracts that were unclear to aid in decision-making. Failure to achieve one or more of the inclusion norms would have meant that the article was disqualified. In case of a need for more clarification or extra data, the specific author was e-mailed. Data extraction was independently achieved by the same two authors (DTA and MYH). A third author (OJ) was consulted to reach a solution when the two authors had disagreements. The data summary tables included the following items: general information (the name of authors, the year of publication, and study setting); methods (study design, treatment comparison); participants (sample size, age, and gender); intervention (the type of interventions, intervention site, and technical aspects of interventions); orthodontic aspects (malocclusion characteristics, type of movement, frequency of orthodontic adjustments, and follow-up time), and outcomes (primary and secondary outcomes mentioned, methods of outcome measurements, the statistical significance of reported differences in patients vs. controls).

Assessment of risk of bias in included studies and strength of evidence

The quality of the selected articles was estimated by two reviewers (DTA and MYH) using Version 2 of the Cochrane risk-of-bias tool for randomized trials (RoB 2) as the included studies were randomized trials [32]. Any conflict was resolved by discussion between the two reviewers. The following domains were evaluated as low, high risk, or some concern of bias for randomized trials: bias arising from the randomization process, bias due to deviations from intended interventions (effect of assignment to intervention; effect of adhering to intervention), bias due to missing outcome data, bias in the measurement of the outcome, and bias in the selection of the reported result. The overall risk-of-bias judgment of the included trials was assessed according to the following: "low risk of bias" if all fields were estimated as "at low risk of bias"; "some concerns" if at least one domain was assessed as "some concerns" but not to be at "high risk of bias" for any domain; "high risk of bias" if at least one or more fields were estimated as "at high risk of bias" or if there were some concerns for multiple domains in a way that substantially lowered confidence in the result. Grading of Recommendations Assessment, Development, and Evaluation (GRADE) approach was used to obtain a supplemental summary of the reliability of the conclusions and strength of the evidence [33] as follows: high, moderate, low, or very low.

Data synthesis and statistical analysis

The details of the electronic search strategy are presented in Table 1.

**Table 1:** Electronic search strategy

Database	Search strategy
CENTRAL (The Cochrane Library)	#1 orthodontic* OR orthodontic tooth movement" OR "orthodontic Treatment" OR "orthodontic Therapy" #2 accelerat* OR rapid* OR short* OR speed* OR fast #3 (surgical OR invasive OR minimally invasive) AND (combine* OR join* OR associate*) AND (multiple or repeat* or duplicat*) #4 (non-surgical OR non-invasive) AND (combine* OR join* OR associate*) AND (multiple or repeat* or duplicat*) #5 #1 AND #2 #6 #3 AND #5 #7 #4 AND #5
EMBASE	#1 orthodontic* OR orthodontic tooth movement" OR "orthodontic Treatment" OR "orthodontic Therapy" #2 accelerat* OR rapid* OR short* OR speed* OR fast #3 (surgical OR invasive OR minimally invasive) AND (combine* OR join* OR associate*) AND (multiple or repeat* or duplicat*) #4 (non-surgical OR non-invasive) AND (combine* OR join* OR associate*) AND (multiple or repeat* or duplicat*) #5 #1 AND #2 #6 #3 AND #5 #7 #4 AND #5
PubMed	#1 orthodontic* OR orthodontic tooth movement" OR "orthodontic Treatment" OR "orthodontic Therapy" #2 accelerat* OR rapid* OR short* OR speed* OR fast #3 (surgical OR invasive OR minimally invasive) AND (combine* OR join* OR associate*) AND (multiple or repeat* or duplicat*) #4 (non-surgical OR non-invasive) AND (combine* OR join* OR associate*) AND (multiple or repeat* or duplicat*) #5 #1 AND #2 #6 #3 AND #5 #7 #4 AND #5
Scopus	#1 TITLE-ABS-KEY (orthodontic* OR "orthodontic tooth movement” OR "orthodontic Treatment" OR "orthodontic Therapy"). #2 TITLE-ABS-KEY (accelerat* OR rapid* OR short* OR speed* OR fast) #3TITLE-ABS-KEY (“surgical” OR “invasive” OR “minimally invasive”) AND (combine* OR join* OR associate*) AND (multiple or repeat* or duplicat*) #4 TITLE-ABS-KEY (“non-surgical” OR “non-invasive”) AND (combine* OR join* OR associate*) AND (multiple or repeat* or duplicat*) #5 #1 AND #2 #6 #3 AND #5 #7 #4 AND #5
Web of Science	#1TS = (orthodontic OR "orthodontic tooth movement” OR "orthodontic Treatment" OR "orthodontic Therapy"). #2TS = (accelerat* OR rapid* OR short* OR speed* OR fast) #3TS = (surgical OR invasive OR minimally invasive) AND TS = (combine* OR join* OR associate*) AND (multiple or repeat* or duplicat*) #4TS = (non-surgical OR non-invasive AND TS = (combine* OR join* OR associate*) AND (multiple or repeat* or duplicat*) #5 #1 AND #2 #6 #3 AND #5 #7 #4 AND #5
Google Scholar	#1 (orthodontic OR "orthodontic tooth movement” OR "orthodontic Treatment" OR "orthodontic Therapy") AND (accelerat* OR rapid* OR short* OR speed* OR fast) AND (surgical OR invasive OR minimally invasive ) AND (combine* OR join* OR associate*) #2 (orthodontic OR "orthodontic tooth movement” OR "orthodontic Treatment" OR "orthodontic Therapy") AND (accelerat* OR rapid* OR short* OR speed* OR fast) AND (non-surgical OR non-invasive) AND (combine* OR join* OR associate*) AND (multiple or repeat* or duplicat*)
Trip	(orthodontic* OR "orthodontic tooth movement” OR "orthodontic Treatment" OR "orthodontic Therapy") AND (accelerate* OR rapid* OR short* OR speed* OR fast) AND (surgical OR invasive OR minimally invasive) AND (combine* OR join* OR associate*) OR (non-surgical OR non-invasive) AND (combine* OR join* OR associate*) AND (multiple or repeat* or duplicate*)
OpenGrey (http://www.opengrey.eu/)	#1 acceleration AND tooth movement #2 orthodontic AND acceleration #3 (surgical OR invasive OR minimally invasive ) AND (combine* OR join* OR associate*) AND (multiple or repeat* or duplicate*) #4 (non-surgical OR non-invasive) AND (combine* OR join* OR associate*) AND (multiple or repeat* or duplicate*)
PQDT OPEN (from proQuest)	#1 acceleration AND tooth movement #2 orthodontic AND acceleration
World Health Organization (WHO) International Clinical Trials Registry Platform (ICTRP) Search Portal	(orthodontic OR ‘tooth movement’ OR ‘orthodontic tooth movement’) AND (accelerate* OR rapid* OR short* OR speed* OR fast)
ClinicalTrials.gov	(orthodontic OR ‘tooth movement’ OR ‘orthodontic tooth movement’) AND (accelerate* OR rapid* OR short* OR speed* OR fast)

Treatment intervention, trial protocol, patients, methodology, and outcome measures were taken into account when evaluating the included studies' heterogeneity. At first, heterogeneity was assessed visually and then mathematically. For conducting a meta-analysis, the RTM following the canine retraction in one month was considered. The meta‑analysis was carried out using Review Manager Version 5.4.1 (The Nordic Cochrane Centre, the Cochrane Collaboration, Copenhagen, Denmark). Mean difference (MD) with a confidence interval of 95% was estimated as the included articles used the same scale for outcome measurements [29]. If I2 was greater than 40%, the heterogeneity was considered as high, and the random effect model was used for meta-analysis [29].

Results

Study Selection and Inclusion in the Review

**Figure 1:**
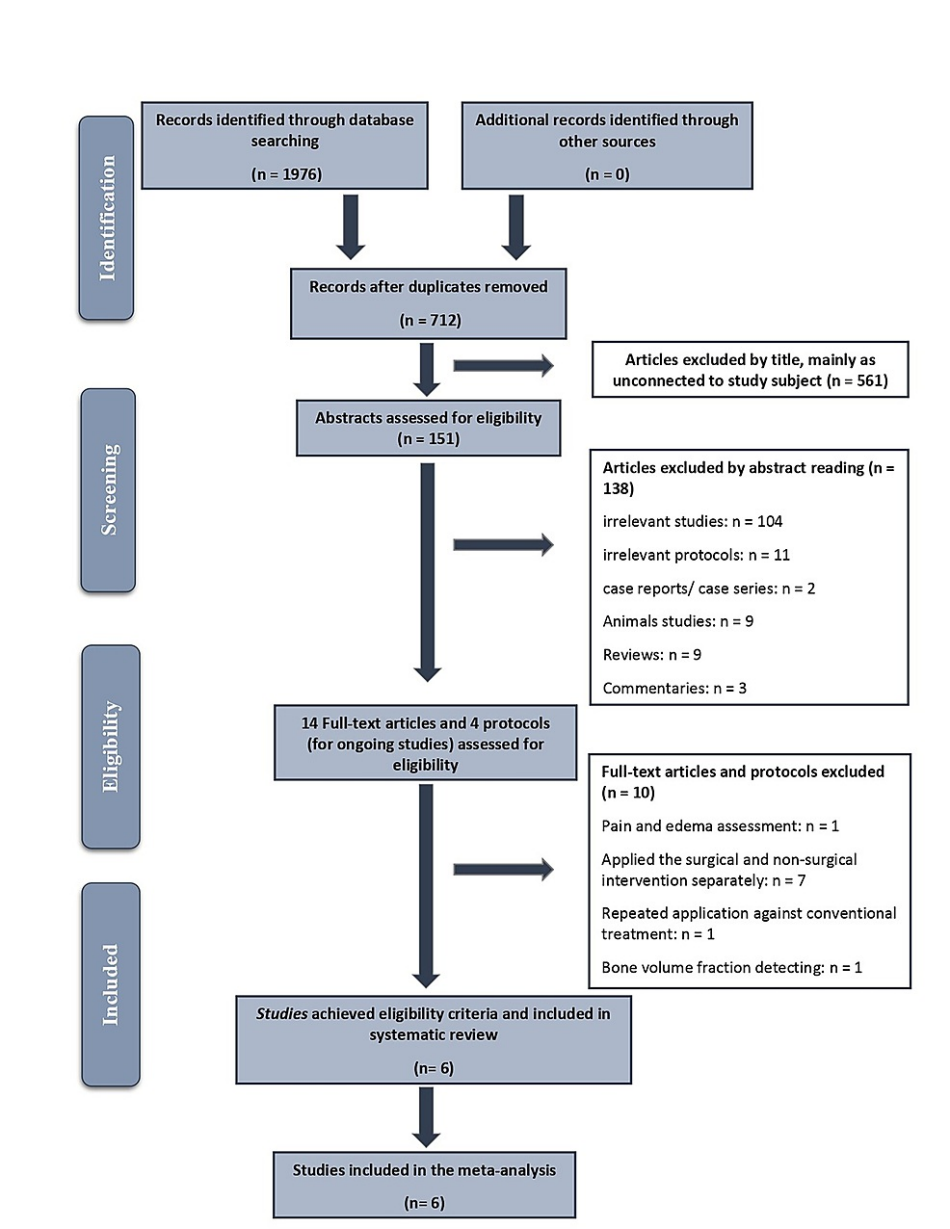
The PRISMA flow diagram of the retrieved studies PRISMA: Preferred Reporting Items for Systematic Reviews and Meta-Analyses

A flow chart of study selection for this review is presented in Figure 1. A total of 1,967 articles were identified from the electronic databases. After taking off duplicates and reviewing titles and abstracts, full texts of 14 potentially relevant papers were evaluated in-depth. Ten completed studies and two of the ongoing studies did not meet the inclusion criteria. A summary of the excluded articles after full-text assessment with reasons for exclusion is illustrated in Table 2. Subsequently, six RCT trials were included in the SR.

**Table 2:** Excluded studies and the reasons for exclusion

Study	Reason for exclusion
Rajasekaran UB, Krishna Nayak US (2014). Effect of prostaglandin E1 versus corticotomy on orthodontic tooth movement: an in vivo study. Indian Journal of Dental Research: official publication of the Indian Society for Dental Research. 25(6):717-21	The study did not apply surgical and non-surgical acceleration methods together, but they were applied separately in the same group of patients
Muñoz F, Jiménez C, Espinoza D, Vervelle A, Beugnet J, Haidar Z (2016). Use of leukocyte and platelet-rich fibrin (L-PRF) in periodontally accelerated osteogenic orthodontics (PAOO): Clinical effects on edema and pain. Journal of Clinical and Experimental Dentistry, 8(2),	A cohort observational study; did not detect the tooth movement acceleration; it used leukocyte and platelet-rich fibrin (L-PRF) in periodontally accelerated osteogenic orthodontics (PAOO) to detect edema and pain
El-Ashmawi N, Abd El-Ghafour M, Nasr S, Fayed M, El-Beialy A, Nasef E (2018). Effect of surgical corticotomy versus low-level laser therapy (LLLT) on the rate of canine retraction in orthodontic patients. Orthodontic Practice US. 9:1-11	The study did not apply surgical and non-surgical acceleration methods together, but they were applied separately in the same group of patients
Haliloglu-Ozkan T, Arici N, Arici S (2018). In-vivo effects of flapless osteopuncture-facilitated tooth movement in the maxilla and the mandible. Journal of Clinical and Experimental Dentistry, 10(8), e761‏	The study compared the repeated application of osteopuncture against conventional orthodontic treatment
Sedky Y, Refaat W, Gutknecht N, ElKadi A (2019). Comparison between the effect of low-level laser therapy and corticotomy-facilitated orthodontics on RANKL release during orthodontic tooth movement: a randomized controlled trial. Lasers in Dental Science. 3(2):99-109	The study did not apply surgical and non-surgical acceleration methods together, but they were applied separately in the same group of patients
Abdarazik MA, Ibrahim SA, Hartsfield JK, AlAhmady HH (2020). The effect of using full-thickness mucoperiosteal flap versus low-level laser application on orthodontic tooth movement acceleration. Al-Azhar Dental Journal for Girls. 7(2 April-Pediatric dentistry and orthodontics issue (Pediatric Dentistry, Orthodontics)):285-93	The study did not apply surgical and non-surgical acceleration methods together, but they were applied separately in the same group of patients
Teh NHK, Sivarajan S, Asif MK, Ibrahim N, Wey MC (2020). Distribution of mandibular trabeculae bone volume fraction in relation to different MOP intervals for accelerating orthodontic tooth movement: A randomized controlled trial. The Angle Orthodontist, 90(6), 774-782‏	The study aimed to investigate the effect of different intervals of micro-osteoperforation on the horizontal and vertical distribution of mandibular trabecular bone volume fraction
Türker G, Yavuz İ, Gönen ZB (2020). Which method is more effective for accelerating canine distalization short term, low-level laser therapy or piezocision? A split-mouth study. Journal of Orofacial Orthopedics/Fortschritte der Kieferorthopädie. :1-9	The study did not apply surgical and non-surgical acceleration methods together, but they were applied separately in the same group of patients
CTRI/2018/05/014328: Comparison of micro-osteoperforation and low-level laser therapy on the rate of retraction-an in vivo study	Ongoing trial (protocol): the study is not applying surgical and non-surgical acceleration methods together, but they are applied separately in the same group of patients
NCT03308851: Evaluation of the effects of osteoperforation and piezocorticision on canine retraction	Ongoing trial (protocol): the study is not applying surgical and non-surgical acceleration methods together, but they are applied separately in the same group of patients

Characteristics of studies

The characteristics of the six included trials [16,34-38] are illustrated in Table 3 and Table 4. Only two trial protocols were found; more information about those ongoing research projects is given in Table 5 and Table 6.

**Table 3:** Characteristics of the included studies: PICOS, follow-up period, and main findings PICOS: patient/population, intervention, comparison, and outcomes; RCT: randomized controlled trial; NAC: non-accelerated control; compound design: consisting of both parallel and split-mouth; MOPs: micro-osteoperforations; LLLT: low-level laser therapy; Exp: experimental; M: male; F: female; RTM: rate of tooth movement; TTM: time of tooth movement

Authors (year, country)	Methods		Participants	Type of Malocclusion	Interventions			Outcomes
	Study design	Treatment comparison	Patients (M/F); age (years)		Type and site of intervention/technical aspects of interventions	Application frequency	Follow-up time	Primary and secondary outcomes
Multiple methods of acceleration (two or more different methods of acceleration)								
Abdelhameed and Refai, 2018, Minya, Egypt [34]	RCT (compound design)	MOPs/NAC vs. LLLT/NAC vs. MOPs + LLLT/NAC	Patients (M/F): 30 (NR/NR). Control: 30. Exp: 30. Age (years): 15-25	Patients who need to extract maxillary 1st premolars and maxillary canine retraction	MOPs: 12 MOPs (with a depth of 6 mm) were applied by miniscrews (six MOPS were done buccally and six palatally). LLLT: a soft laser (wavelength: 810 ± 10 nm) was used from buccal and palatal surfaces along the root of the U3	MOPs: the technique was repeated every two weeks. LLLT: the application of laser was at the beginning of a canine retraction, after three days, one week, two weeks, then every two weeks for three months	3 months	Primary outcome: RTM (mm/week)
Farid et al., 2019, Cairo, Egypt [36]	RCT (split-mouth design)	Corticotomy + LLLT vs. corticotomy	Patients (M/F): 16 (0/16). Control: 16. Exp: 16. Age (years): 17-25	Class I or Class II (Angles’ classification) malocclusion cases needed to extract 1st premolars	Corticotomy: after an elevated full-thickness flap, 10-15 corticotomy perforations with a depth of 1-2 mm were done from the distal surface of the 2nd premolar to the mesial surface of the U3, using a round bur. LLLT: InGaAs diode laser (wavelength: 940 ± 10 nm) was applied at the middle point of the U3 root on buccal and palatal surfaces for 240 seconds	LLLT: the application of laser was on the 1stday of retraction, after one, two, and three weeks, then every two weeks. The application of LLLT started on the same day of surgery	4 months	Primary outcome: RTM (mm/month). Secondary outcomes: molar anchorage loss
Yousif et al., 2019, Tanta, Egypt [16]	RCT (compound design)	Multiple osteoperforation/NAC vs. multiple osteoperforation + corticotomy/NAC	Patients (M/F): 30 (NR/NR). Control: 30. Exp: 30. Age (years): 15-18	Patients who need to extract 1st premolars and maxillary canine retraction	Multiple osteoperforation: after an elevated flap, 3 MOPs (2-mm wide, 2-mm deep, and 2 mm apart from each other) were done along the mesial and distal side of the U3 root, using round surgical bur. Corticotomy: after an elevated flap, a corticotomy cut along the distal side of the U3 root was carried out		Until the completion of the canine retraction	Primary outcome: TTM (days). Secondary outcomes: pain and discomfort., canine angulation
Repetition of an acceleration method								
Sivarajan et al., 2019, Kuala Lumpur, Malaysia [38]	RCT (compound design)	MOP 4-weekly maxilla/8-weekly mandible/NAC vs. MOP 8-weekly maxilla/12-weekly mandible/NAC vs. MOP 12-weekly maxilla/4-weekly mandible/NAC	Patients (M/F): 30 (7/23). Control: 30. Exp: 30. Age (years): 18 years and above	Patients who need to extract four first premolars and canine retraction	MOPs: 3 MOPs (with a depth of 3 mm and 2 mm apart from each other vertically) were applied using an Orlus screw (through the buccal mucosa adjacent to the extraction site)	MOPs: the technique was repeated every 4 weeks in Group 1 (4 sessions of MOPs), 8 weeks in Group 2 (2 sessions of MOPs), and 12 weeks in Group 3 (2 sessions of MOPs)	4 months	Primary outcome: RTM (mm/month). Secondary outcomes: pain and its impact on daily function
Asif et al., 2020, Kuala Lumpur, Malaysia [35]	RCT (compound design)	MOP 4-weeks/NAC vs. MOP 8-weeks/NAC vs. MOP 12-weeks/NAC	Patients (M/F): 30 (NR/NR). Control: 30. Exp: 30. Age (years): 18 years and above	Patients who need to extract four first premolars and canine retraction	MOPs: 3 MOPs (with a depth of 3 mm and 2 mm apart from each other vertically) were applied using an Orlus screw (through the buccal mucosa of the extraction site)	MOPs: the technique was repeated every 4 weeks in Group 1 (4 sessions of MOPs), 8 weeks in Group 2 (2 sessions of MOPs), and 12 weeks in Group 3 (2 sessions of MOPs)	3 months	Primary outcome: RTM (mm/month)
Jaiswal et al., 2021, New Delhi, India [37]	RCT (split-mouth design)	One-time MOP vs. two-time MOP	Patients (M/F): 16 (4/13). Control: 16. Exp: 16. Age (years): 15-25	Patients who need to extract 1st premolars and maxillary canine retraction	MOPs: 3 MOPs (with a depth of 7 mm) were applied using Propel (through the buccal mucosa of the extraction site)	MOPs: the technique was repeated one month after the first MOP in the Exp Group	6 months or until the completion of the canine retraction	Primary outcome: RTM (mm/month), molar anchorage loss, canine angulation

**Table 4:** (Continuation of Table 3): Additional Characteristics of the included studies (appliance and anchorage used, orthodontic adjustments, statistical outcomes, and methods of primary outcome measurements) TADs: temporary anchorage devices; SS: stainless steel; U3: upper canines; L3: lower canines; TPA: trans-palatal arch: RTM; rate of tooth movement; TTM: time of tooth movement; MOPs: micro-osteoperforations; LLLT: low-level laser therapy; 5-PLS: 5-point Likert scale; VAS: visual analog scale; IOPA: intraoral periapical radiographs

Authors (Year, Country)	Appliance characteristics	Anchorage used	Orthodontic adjustments	Statistical significance of reported outcomes	Methods of primary outcome measurements
				Primary and secondary outcomes	
Multiple methods of acceleration (two or more different methods of acceleration)					
Abdelhameed and Refai, 2018, Minya, Egypt [34]	MBT prescription brackets + NiTi closed-coil springs (150 g) for retraction U3	TADs between 5 and 6	Every two weeks	RTM (mm/week): 2nd, 4th, and 6th week: (MOPs) p-value = 0.000 8th, 10th, 12th week: (MOPs) p-value = 0.001 2nd, 4th, 6th, 8th, 10th, 12th week: (LLLT) p-value = 0.001 2nd, 4th, 6th, 8th, 10th, 12th week: (MOPs and LLLT) p-value = 0.000 2nd week: MOPs: 1.3 ± 0.12/LLLT: 0.98 ± 0.27/MOPs and LLLT: 1.82 ± 0.19 4th week: MOPs: 2.16 ± 0.27/LLLT: 1.81 ± 0.39/MOPs and LLLT: 2.83 ± 0.12 6th week: MOPs: 2.92 ± 0.73/LLLT: 2.38 ± 0.27/MOPs and LLLT: 3.46 ± 0.64 8th week: MOPs: 3.43 ± 0.66/LLLT: 2.63 ± 0.87/MOPs and LLLT: 3.86 ± 0.27 10th week: MOPs: 3.92 ± 0.88/LLLT: 3.26 ± 0.89/MOPs and LLLT: 4.39 ± 0.73 12th week: MOPs: 4.33 ± 0.64/LLLT: 3.72 ± 0.71/MOPs and LLLT: 4.87 ± 0.88	Direct intraoral measurements using a digital intraoral caliper
Farid et al., 2019, Cairo, Egypt [36]	Roth prescription brackets + 0.017 x 0.025-inch SS + NiTi closed-coil springs (150 g) for retraction U3	Soldered TPA	Every two weeks	RTM (mm/week): 1stmonth: p-value = 0.019 Corticotomy + LLLT: 0.81 ± 0.58, Corticotomy: 1.16± 0.67 2nd month: p-value = 0.064 Corticotomy + LLLT: 1.04 ± 0.61, Corticotomy: 0.82 ± 0.36 3rdmonth: p-value = 0.968 corticotomy + LLLT: 1.83 ± 1.00, Corticotomy: 2.01 ± 1.37 4thmonth: p-value = 0.033 Corticotomy + LLLT: 1.43 ± 1.18, Corticotomy: 0.83± 1.03	Measurements were done using 3D-scanned study models
Yousif et al., 2019, Tanta, Egypt [16]	Roth prescription brackets + 0.016 x 0.022-inch SS + elastomeric chain for retraction U3, giving force (150 g) that was replaced every three days	Soldered TPA	Every week	TTM (days): p-value = 0.001 MOPs: 11.0 ± 2.36 Corticotomy + MOPs: 15.2 ± 1.62 Control: 8.1 ± 1.90 Canine angular changes: p-value = 0.001 Multiple osteoperforations: 11.0 ± 2.36 corticotomy + multiple osteoperforations: 67.7 ± 3.09 Control: 110.5 ± 4.84 Acceleration rate: multiple osteoperforations, corticotomy + multiple osteoperforations accelerated the canine retraction by 22%, 38.5%, respectively	Direct intraoral measurements using a digital intra-oral caliper. Canine angulation was assessed by panoramic radiography
Repetition of an acceleration method					
Sivarajan et al., 2019, Kuala Lumpur, Malaysia [38]	MBT prescription brackets (0.022x 0.028-inch slot) + 0.018 x 0.025-inch SS + elastomeric chain (140-200 g) for retraction U3 and L3	TADs between 5 and 6	Every month	RTM (mm/4 months): p-value = 0.004 MOP-4: 3.96 ± 1.71 MOP-8: 4.15 ± 1.71 MOP-12: 4.39 ± 1.78 Control: 3.06 ± 1.64 Pain and its impact on daily function: MOP-4: 3.96 ± 1.71 moderate (score 2/5), 60% of patients severe (score 3/5), 15% of patients MOP-8: 1.35 ± 0.59 mild (score 1/5), 70% of patients MOP-12: 1.3 0± 0.57 mild (score 1/5), 75% of patients	Direct intraoral measurements using a digital intraoral caliper. Pain intensity was assessed by 5-PLS, whereas VAS was used to assess its impact
Asif et al., 2020, Kuala Lumpur, Malaysia [35]	MBT prescription brackets (0.022 x 0.028-inch slot) + 0.018 x 0.025-inch SS + elastomeric chain (140-200 g) for retraction L3	TADs between 5 and 6	Every month	RTM (mm/3 months): p-value:<0.001 MOP: 4.03 ± 0.79, Control: 2.77 ± 0.79 p-value = 0.001 MOP-4: 4.57 ± 0.77, Control: 3.08 ± 0.77 p-value = 0.006 MOP-8: 3.06 ± 0.60, Control: 1.94 ± 0.60 p-value = 0.004 MOP-12: 4.17 ± 0.92, Control: 3.03 ± 0.92	Direct intraoral measurements using a digital intraoral caliper
Jaiswal et al., 2021, New Delhi, India [37]	Roth prescription brackets (0.022 slot) + 0.019 x 0.025-inch SS + NiTi closed-coil springs (150g) for retraction U3	TADs between 5 and 6	Every month	RTM (mm/month): 1stmonth: p-value = 0.840 One-time MOP: 1.37 ± 0.43, Two-time MOP: 1.41 ± 0.43 2nd month: p-value<0.001 One-time MOP: 2.40 ± 0.52, Two-time MOP: 3.20 ± 0.64 3rdmonth: p-value<0.001 One-time MOP: 3.31 ± 0.87, Two-time MOP: 4.68 ± 1.01 6th month: p-value<0.001 One-time MOP: 4.57 ± 0.54, Two-time MOP: 6.12 ± 0.76 Canine angular changes: p-value = 0.001 1st month: p-value = 0.907 One-time MOP: 97.13 ± 9.2, Two-time MOP: 97.13 ± 8.7 2ndmonth: p-value = 0.889 One-time MOP: 96.31 ± 9.09, Two-time MOP: 95.88 ± 8.56 3rdmonth: p-value = 0.727 One-time MOP: 95.13 ± 8.90, Two-time MOP: 94.06 ± 8.11 Molar anchorage loss: p-value = 0.657 One-time MOP: 0.31 ± 0.24, Two-time MOP: 0.30 ± 0.39	Measurements were done using 3D scanned study models. Canine angulation was assessed by IOPA

**Table 5:** Protocols of the ongoing studies registered at the WHO ICTRP WHO ICTRP: World Health Organization International Clinical Trials Registry Platform; RCT: Randomized controlled trial, U3: upper canine, NR: not reported; TTM: time of tooth movement; RTM: rate of tooth movement

Study ID	Trial name or title	Study design	Intervention + treatment comparison	Sample size/age/gender	Outcomes
CTRI/2018/07/015109	Effectiveness of combined piezocision and low-level laser therapy in reducing orthodontic treatment duration and patient discomfort: A randomized controlled trial	RCT	Piezocision and low-level laser therapy versus conventional orthodontic treatment	17/NR/NR	Primary outcomes: TTM. Secondary outcomes: the analgesic effect of low-level laser therapy following piezocision
CTRI/2020/04/024453	Effectiveness of piezocision-assisted corticotomy and low-level laser therapy in enhancing rapid maxillary canine retraction: A randomized controlled trial	RCT	Piezocision-assisted corticotomy versus low-level Laser therapy (LLLT) versus LLLT and piezocision versus control	40/NR/NR	Primary outcomes: RTM. Secondary outcomes: molar anchorage loss, the periodontal index for the U3, and canine vitality and root resorption

**Table 6:** Additional characteristics of the protocols of ongoing studies NR: not reported; U3: upper canine, LLLT: low-level laser therapy

Study ID	Setting	Orthodontic aspects	Technical aspects of interventions	Notes
CTRI/2018/07/015109	Department of Orthodontics and Dentofacial Orthopaedics, Manipal College of Dental Sciences, India	Baseline Characteristics: subjects requiring maxillary canine retraction following 1st premolar extraction as a part of their treatment plan. Subjects with permanent dentition. No prior H/o orthodontic treatment	Piezocision: two vertical cuts mesial and distal of the U3, 5-8 mm long, 3mm deep, and sutured if necessary. LLLT: a semiconductor (GaAs) diode with a wavelength of 980 nm, and total energy of 2.5 J, for the 10 points along the root of the maxillary canine	This study is currently in Phase 3. Starting date: 01-08-2018. Completion date: NR
CTRI/2020/04/024453	Department of Orthodontics and Dentofacial Orthopedics, Teerthankar Mahaveer, Moradabad, India	Baseline Characteristics: patients requiring first upper premolars extraction and two-step retraction technique. Complete permanent dentition (except third molars). No previous orthodontic treatment. Healthy patients without systemic diseases that can affect bone and tooth movement. Good oral hygiene and healthy periodontium, which will be evaluated clinically	NR	This study is not yet recruiting. Starting date: 15-04-2020. Completion date: NR

Six completed RCTs [16,34-38], with a total of 152 patients, with ages ranging from 15 to 25 years, were included in this SR. One study included only female patients [36], three studies did not give information about sex distribution [16,34,35], while the other two studies included both genders, with a predominance of females [37,38]. Four of the involved studies were of a compound design (COMP) [a parallel-group design with a split-mouth design (SMD) in each group] [16,34,35,38], and the others were of SMD [36,37]. Three studies touched on the multiplicity of acceleration methods. Two of them combined LLLT and surgical interventions (MOPs, corticotomy) [34,36]. In one paper, the authors evaluated LLLT + MOPs versus each of these techniques separately [34], whereas, in the other study, only the comparison between LLLT + corticotomy versus corticotomy was performed [36]. Moreover, the third study combined multiple osteoperforation with a corticotomy procedure against multiple osteoperforation only [16]. On the other hand, the other three studies [35,37,38] discussed the efficacy of repeating the acceleration procedures. All three papers dealt with the repetition of MOPs at different time intervals. The surgical interventions in the retrieved studies ranged from invasive (traditional corticotomy [16,36], multiple osteoperforation with flap elevation [16]) to minimally invasive (MOPs [34,35,37,38]).

All the included studies [16,34-38] involved extraction‑based treatments and were primarily about accelerating canine retraction. Four papers studied the upper canines retraction [16,34,36,37], one studied the distalization of both upper and lower canines [38], and one trial assessed the retraction of lower canines [35]. The retraction was performed on canines after the first premolar extraction, which was performed before the beginning of orthodontic treatment in two papers [34,37], and after the completion of leveling and alignment in two trials [16,36], while this information was not mentioned in the other studies [35,38].

Measurement of tooth movement was expressed as RTM in five papers [34-38], and as TTM in one paper [16]. Four studies depended on temporary anchorage devices (TADs) [34,35,37 38], whereas the other studies used soldered trans-palatal arch for anchorage [16,36]. Concerning the method used to measure the speed of tooth movement, there were differences between the trials. Four of the included studies used digital intraoral caliper [16,34,35,38], whereas the others conducted the measurements using 3D-scanned study models [36,37]. Follow-ups varied between three months [34,35], to four months [36,38], and till the completion of canine retraction [16,37].

Risk of bias of the included studies

**Figure 2:**
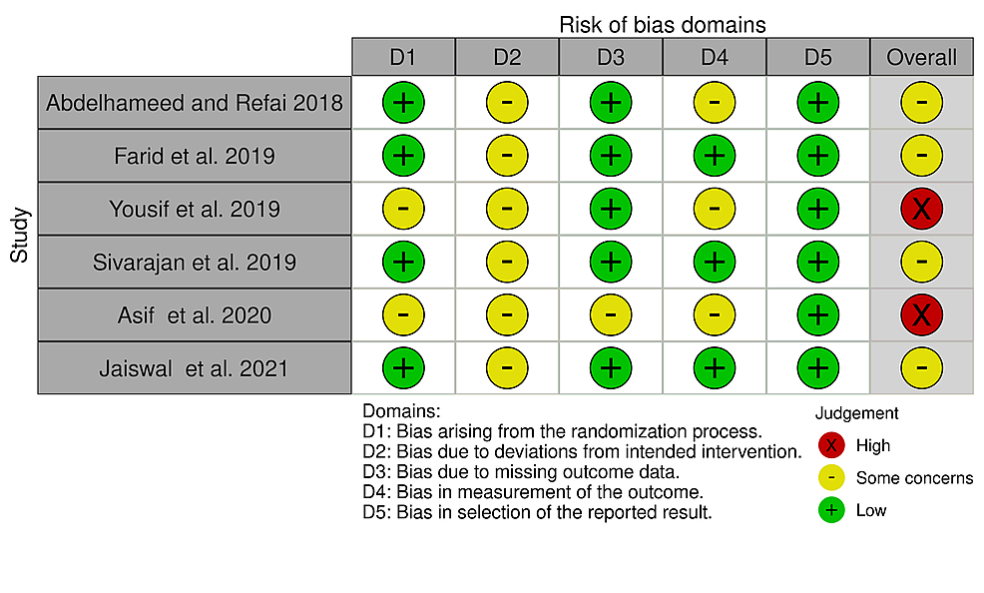
Risk of bias summary of RCTs + sign: low risk of bias; - sign: some concern of bias; X sign: high risk of bias RCTs: randomized controlled trials

**Figure 3:**
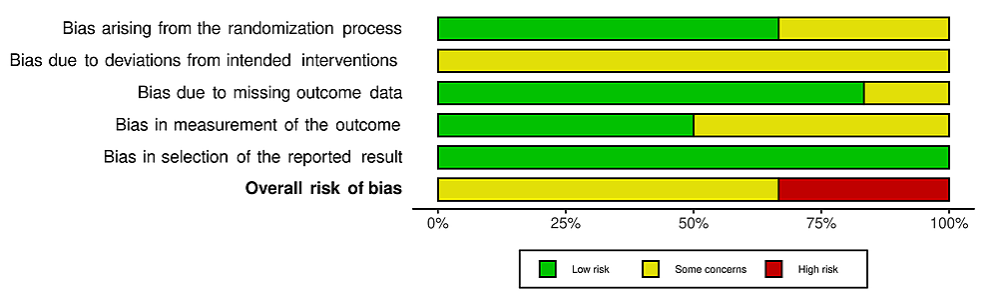
The overall risk of bias score for each field of RCTs RCTs: randomized controlled trials

The risk of bias of the included trials is demonstrated in Figure 2, while Figure 3 shows the overall risk of bias for each field. More details about the assessment of the bias risk with supporting reasons for every assessment can be found in Table 7. Four of the included studies [34,36-38] were assessed as "some concern of bias", but the other two trials [16,35] were at 'high risk of bias". Bias due to deviations from intended interventions (effect of assignment to intervention or effect of adhering to intervention) and bias in the measurement of the outcomes were the most questionable domains (100% of some concern of bias in all studies, and 50% in three studies, respectively). 

**Table 7:** Risk of bias assessment according to RoB 2 tool RoB 2: Cochrane risk-of-bias tool for randomized trials

Study	Bias arising from the randomization process	Bias due to deviations from intended interventions		Bias due to missing outcome data	Bias in measurement of the outcome	Bias in selection of the reported results
		Effect of assignment to interventions	Effect of adhering to interventions			
Abdelhameed and Refai, 2018 [34]	Low risk: "assignment of patients and the sides of interventions were performed as follows: computer-generated random numbers were done using Microsoft Office Excel 2013 sheet". (Page 2181)	Some concerns: blinding cannot be performed. There is "no information" on whether any deviations arose because of the trial context	Some concerns: blinding cannot be performed. And "no information" on whether the important non-protocol interventions were balanced across intervention groups	Low risk: "during the study, there was one dropout patient in Group C. Also, there were some missing appointments which were all recorded as follows: Group (A), two missing patient appointments at the 4th, and 10th weeks. Group (B), one missing patient appointment in the 10th week. Group (C), no missing patient appointments but there was one dropout patient as mentioned previously". (Page 2182). "Nearly all" the outcome data is available.	Some concerns: the method of measuring the outcome is appropriate but the outcome assessor was not blind for the assignment of each intervention. "Data for the evaluation of each intervention were collected by direct intraoral measurements using a digital intraoral caliper". (Page 2182)	Low risk: the numerical result being assessed has not probably been selected, based on the results, from multiple eligible outcome measurements within the outcome domain and analyses of the data. The eligible reported results for the outcome corresponded to the intended outcome measurements
Farid et al., 2019 [36]	Low risk: "random numbers were generated on a computer using Microsoft Office Excel 2007 sheet by a person who was not involved in the clinical trial (MA). The concealed allocation was performed by using a set of random numbers placed in sealed opaque envelopes. Each patient picked up a number that would represent the intervention side (laser + corticotomy) performed either on the RT side or the LT side and thus the opposing number would be the comparator side (corticotomy only). By calling FS who was accessible to the random table, the intervention which will be performed either on the LT or the RT side was revealed. At the time of intervention, the subject was allowed to choose one of the envelopes to detect her number in the randomization sequence and thus detect which was the intervention side". (Page 276)	Some concerns: blinding cannot be performed. There is "no information" on whether any deviations arose because of the trial context	Some concerns: blinding cannot be performed. And "no information" on whether the important non-protocol interventions were balanced across intervention groups	Low risk: "all patients had successfully completed the four months duration of the study except for 3 dropout patients who did not continue the follow-up visits at the beginning of leveling and alignment phase of the orthodontic treatment that was substituted by another 3 patients who were fulfilling the same inclusion criteria of the study." "During the course of the study, there were no losses in the pre-intervention or in the final records derived from the dental models. No dropout visits were recorded regarding the monthly impression visits. As for laser visits, nine patients missed their appointments in the third and fourth months that was recorded by date and was replaced by another consecutive visit." (Page 279)	Low risk: the method of measuring the outcome is appropriate and the outcome assessor was blind for the assignment of each intervention. "Three-dimensional digital models were obtained by scanning the sequential stone models using a surface laser scanner. The incremental rate of canine retraction was then measured using a 3-shape program". (Page 278). "Landmark identification was done through two blinded assessors (NA and AN) and an average of their measurements was considered for the statistical analysis". (Page 279)	Low risk: The numerical result being assessed has not probably been selected, based on the results, from multiple eligible outcome measurements within the outcome domain and analyses of the data. The eligible reported results for the outcome corresponded to the intended outcome measurements
Yousif et al., 2019 [16]	Some concerns: the method used for randomization was not reported. “A randomized split-mouth clinical multi-operator study was performed on 30 orthodontic patients". "Subjects were randomized equally into three canine retraction groups"	Some concerns: blinding cannot be performed. There is "no information" on whether any deviations arose because of the trial context	Some concerns: blinding cannot be performed. And "no information" on whether the important non-protocol interventions were balanced across intervention groups	Low risk: "no information" on whether the outcome available for all, or nearly all, participants, and probably the result was not biased by missing outcome data. "Study was carried out to overcome attrition bias (patient dropout) due to poor oral hygiene or bad patient compliances" (Page 3223)	Some concerns: the method of measuring the outcome is appropriate but the outcome assessor was not blind for the assignment of each intervention. "The distance between the distal surface of the canine and the mesial surface of the second premolar was recorded directly in patient’s mouth every week using a caliper with 0.01-mm scale". (Page 3226)	Low risk: the numerical result being assessed has not probably been selected, based on the results, from multiple eligible outcome measurements within the outcome domain and analyses of the data. The eligible reported results for the outcome corresponded to the intended outcome measurements
Sivarajan et al., 2019 [38]	Low risk: "randomized block sampling was carried out using RANDOM.ORG online software to allocate participants into three intervention groups on a 1:1:1 basis". (Page 185)	Some concerns: blinding cannot be performed. There is "no information" on whether any deviations arose because of the trial context	Some concerns: blinding cannot be performed. And "No information" on whether the important non-protocol interventions were balanced across intervention groups	Low risk: "thirty subjects were enrolled into the study between September 2014 and March 2016 with data collection complete by March 2017 and no dropouts". (Page 186). "All" the outcome data is available	Low risk: "the distance from the central point of the canine bracket to the superior margin of the mini implant (maxilla) and the inferior margin of the mini implant (mandible) and the distance from the canine cusp tip to the mesiobuccal groove of the first molar was clinically measured using electric digital calipers (accurate to 0.01 mm)". (Page 185). "The outcome measurements were also blinded". (Page 185). The method of measuring the outcome is appropriate and the outcome assessor was blind for the assignment of intervention	Low risk: the numerical result being assessed has not probably been selected, based on the results, from multiple eligible outcome measurements within the outcome domain and analyses of the data. The eligible reported results for the outcome corresponded to the intended outcome measurements
Asif et al., 2020 [35]	Some concerns: the method used for randomization was not reported. "This study was a single-center, single-blind, prospective randomized split-mouth clinical trial". (Page 580)	Some concerns: blinding cannot be performed. There is "no information" on whether any deviations arose because of the trial context	Some concerns: blinding cannot be performed. And "No information" on whether the important non-protocol interventions were balanced across intervention groups	Some concerns: 6 patients (2 from MOP 4-weeks Group and 4 from MOP 8-weeks Group) were dropouts. The reasons are illustrated in Figure 4 (consort flow diagram). (Page 583)	Some concerns: "the distance of canine movement was recorded every 4 weeks with digital calipers accurate to 0.01 mm, for 12 weeks". (Page 581). “Two observers (orthodontic postgraduate students) were blinded to the frequency of MOP while analyzing the BV/TV ratio using CT analyzer software as CBCT files were labeled by random numbers". (Page 581). The method of measuring the outcome is appropriate, but there is "no information" on whether the outcome assessor was blind to the assignment of each intervention	Low risk: the numerical result being assessed has not probably been selected, based on the results, from multiple eligible outcome measurements within the outcome domain and analyses of the data. The eligible reported results for the outcome corresponded to the intended outcome measurements
Jaiswal et al., 2021 [37]	Low risk: "random numbers were generated in the permuted random block size of 2 using the Research Randomizer software (Research Randomizer, Version 4, Urbaniak GC and Plous S) by the investigator AJ. The numbers were concealed in opaque envelopes and kept in a box. Each patient was then asked to pick a sealed envelope to assign the second MOP to either the right or left side, executed separately without any role of primary clinical investigators, shuffled every time before picking". (Page 417)	Some concerns: blinding cannot be performed. There is "no information" on whether any deviations arose because of the trial context	Some concerns: blinding cannot be performed. And "No information" on whether the important non-protocol interventions were balanced across intervention groups	Low risk: "one patient was excluded after intervention owing to miniscrew implant failure". (Page 418). "Nearly all" outcome data is available	Low risk: “the models were scanned with Maestro 3D scanner (MDS 400, AGE solutions S.r.l., Pisa, Italy) with an accuracy of 0.01 mm to obtain digital models. These digital models were imported in Dolphin 3D software (Version 11.9, Patterson Inc., Chatsworth, CA) and the baseline (T0) model was superimposed on T30, T60, T90, and T180 day models with medial 2/3rd of third rugae as the reference point". (Page 417). “However, the extracted data was coded during collection and analysis to ensure blinding". (Page 417). The method of measuring the outcome is appropriate and the outcome assessor was blind to the assignment of each intervention	Low risk: the numerical result being assessed has not probably been selected, based on the results, from multiple eligible outcome measurements within the outcome domain and analyses of the data. The eligible reported results for the outcome corresponded to the intended outcome measurements

Effects of interventions

Primary outcomes: first theme: multiple methods of acceleration

Combining LLLT With a Surgical Intervention Against the Application of Each Technique Separately

Abdelhameed and Refai [34] estimated the impact of combined LLLT + MOPs versus the impact of the application of each technique separately on the RTM in a three-arm compound-design RCT. When comparing the accelerated sides with the non-accelerated sides, MOPs and LLLT, as well as MOPs + LLLT interventions, showed an increased rate of upper canine retraction. Statistically significant differences were found at all assessment times (p<0.05). On the other hand, the combination of ' MOPs + LLLT was found to be superior to the single application of MOPs or LLLT separately. According to GRADE, the overall quality of evidence supporting this outcome is low (Table 8). The mean rate of canine retraction in the MOPs + LLLT side was the highest in the first, second, and third months (x ®= 2.83 ± 0.12 mm, x ®= 3.86 ± 0.27 mm, x ®= 4.87 ± 0.88 mm, respectively). Moreover, In the first, second, and third months, the mean rate of canine movement in MOPs side (x ®= 2.16 ± 0.27 mm, x ®= 3.43 ± 0.66 mm, x ®= 4.33 ± 0.64 mm, respectively) was significantly greater than the LLLT side (x ®= 1.81 ± 0.39 mm, x ®= 2.63 ± 0.87 mm, x ®= 3.72 ± 0.71 mm, respectively). The MOPs technique accelerated the canine retraction rate by 1.6 fold whereas LLLT achieved this by 1.3 fold compared with the non-accelerated sides. Nevertheless, the combination of MOPs + LLLT gained priority by increasing the canine retraction rate by 1.8 fold compared with the non-accelerated side.

**Table 8:** Summary of findings according to the GRADE guidelines for the included trials High quality: Further research is very unlikely to change our confidence in the estimate of effect. Moderate quality: Further research is likely to have an important impact on our confidence in the estimate of effect and may change the estimate. Low quality: Further research is very likely to have an important impact on our confidence in the estimate of effect and is likely to change the estimate. Very low quality: We are very uncertain about the estimate CI: confidence interval; SP: split-mouth design; COMP: compound design; LLLT: low-level laser therapy MOPs: micro-osteoperforations ^a, b^Decline in one level for risk of bias (bias due to deviations from intended interventions [35,38], bias arising from the randomization process, bias in the measurement of the outcome, and bias in the measurement of the outcome [35]), one level for indirectness**, and one level for imprecision*** ^c, h, j^Decline in one level for risk of bias (bias due to deviations from intended interventions), one level for imprecision*** [37] ^d^Decline in one level for risk of bias (bias due to deviations from intended interventions, bias in the measurement of the outcome), one level for indirectness**, and one level for imprecision*** [35] ^e, g^Decline in one level for risk of bias (bias due to deviations from intended interventions), one level for imprecision*** [36] ^f, g^Decline in one level for risk of bias (bias arising from the randomization process, bias due to deviations from intended interventions, bias in the measurement of the outcome), one level for indirectness**, and one level for imprecision [16] *Differences in results; **Outcome is not directly related; ***Limited number of trials

Quality assessment criteria						Summary of findings				Comments
Number of studies	Risk of bias	Inconsistency	Indirectness	Imprecision	Other considerations	Number of patients	Effects		Certainty	
							Absolute (95% CI)	Relative (95% CI)		
Rate of canine retraction accelerated by multiple MOPs (every 4 weeks versus every 8 weeks)										
2 RCTs (COMP)	Serious	Not serious	Serious	Serious	None	34	-	Relative effect (95% CI): MD 0.24 (-0.28-0.77)	Very low ⊕⊖⊖⊖^a^	
Rate of canine retraction accelerated by multiple MOPs (every 4 weeks versus every 12 weeks)										
2 RCTs (COMP)	Serious	Not serious	Serious	Serious	None	40	-	Relative effect (95% CI): MD 0.06 (-0.14-0.27)	Very low ⊕⊖⊖⊖^b^	
Rate of canine retraction accelerated by multiple MOPs (2 times versus 1 time)										
1 RCT (SP)	Serious	Not serious	Not serious	Serious	None	16	-	Relative effect (95% CI): not estimable	Low ⊕⊕⊖⊖^c^	Application two-time MOP was more efficient than the one-time MOP (p<0.001)
Rate of upper canine retraction accelerated by combined techniques and a single application of technique										
1 RCT (COMP)	Serious	Not serious	Serious	Serious	None	30	-	Relative effect (95% CI): not estimable	Very low ⊕⊖⊖⊖^d^	The combination of MOPs + LLLT is superior to the application of each technique separately
1 RCT (SP)	Serious	Not serious	Not serious	Serious	None	16	-	Relative effect (95% CI): not estimable	Low ⊕⊕⊖⊖^e^	The combination of corticotomy + LLLT was not more efficient than the application of corticotomy only
Time of upper canine retraction accelerated by combined techniques and a single application of technique										
1 RCT (COMP)	Serious	Not serious	Serious	Serious	None	30	-	Relative effect (95% CI): not estimable	Very low ⊕⊖⊖⊖^f^	Canine retraction took more time in multiple osteoperforations side than multiple osteoperforations + corticotomy side
Adverse effects: anchorage loss										
1 RCT (SP)	Serious	Not serious	Not serious	Serious	None	16	-	Relative effect (95% CI): not estimable	Low ⊕⊕⊖⊖^g^	Anchorage loss was greater on the corticotomy side than on the LLLT + corticotomy side
1 RCT (SP)	Serious	Not serious	Not serious	Serious	None	16	-	Relative effect (95% CI): not estimable	Low ⊕⊕⊖⊖^h^	There was an insignificant difference in loss of anchorage between the one-time MOP side and the side of the two-time MOP (p<0.05)
Adverse effects: canine angulation										
1 RCT (COMP)	Serious	Not serious	Serious	Serious	None	30	-	Relative effect (95% CI): not estimable	Very low ⊕⊖⊖⊖^i^	Distal tipping and buccal inclination of canine were greater in the multiple osteoperforation + corticotomy side than the multiple osteoperforation side
1 RCT (SP)	Serious	Not serious	Not serious	Serious	None	16	-	Relative effect (95% CI): not estimable	Low ⊕⊕⊖⊖^j^	There was an insignificant difference in canine tipping between the one-time MOP side and the side of the two-time MOP (p<0.05)

Combining LLLT With a Surgical intervention Against a Single Application of the Latter

Farid et al. [36] evaluated the combined effect of LLLT + corticotomy versus corticotomy only on the RTM in a split-mouth RCT. In the first month of upper canine retraction, the results were opposite of what was expected. The mean rate of canine movement was greater in corticotomy only side (x ®= 1.16± 0.67 mm) compared with the LLLT + corticotomy side (x ®= 0.81± 0.58 mm) with a statistically significant difference (p = 0.019). Conversely, the mean rate of canine movement in the fourth month was greater in the LLLT + corticotomy side (x ®= 1.43± 1.18 mm) than corticotomy only side (x ®= 0.83± 1.03 mm) with a statistically significant difference (p = 0.033). However, there was no statistically significant difference between the sides of LLLT + corticotomy and corticotomy only regarding the rate of upper canine retraction in the second and third months (p = 0.064, p = 0.968), respectively. The high heterogeneity between the previous studies [34,36] did not allow for conduct quantitative synthesis of the findings.

Combining Two Different Surgical Interventions Against a Single Application of One of Them 

The clinical trial by Yousif et al. [16] evaluated the effect of the combined multiple osteoperforation + corticotomy procedure against multiple osteoperforation on TTM in a three-arm compound-design RCT. The multiple osteoperforation side required less than three months (x ®= 85.1 ± 3.03 days) for the completion of canine retraction, whereas the multiple osteoperforation + corticotomy side took about two months only (x ®= 67.7 ± 3.09 days) to complete this procedure, with a statistically significant difference between the two sides (p = 0.001). According to GRADE, the overall quality of evidence supporting this outcome is low (Table 8). The canine retraction was accelerated by 22% for the multiple osteoperforation side and 38.5% for the multiple osteoperforation + corticotomy side. Due to the use of a different outcome variable between this study and the previous, the meta-analyses were not performed.

Second theme: repetition of an acceleration method

**Figure 4:**
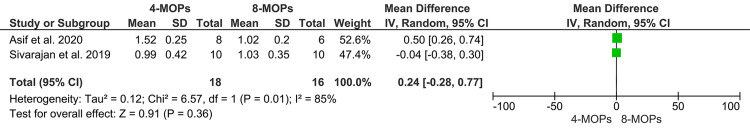
Forest plot of the comparison between the four-weekly MOPs and eight-weekly MOPs of canine retraction in one month MOP: micro-osteoperforation; CI: confidence interval

**Figure 5:**
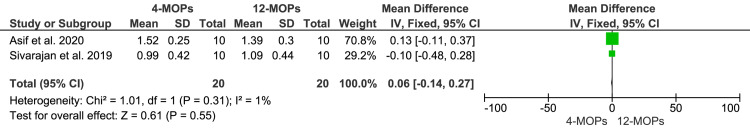
Forest plot of the comparison between the four-weekly MOPs and 12-weekly MOPs of canine retraction in one month MOP: micro-osteoperforation; CI: confidence interval

Three of the included studies assessed the repetition of acceleration procedures. Moreover, all of these studies focused on the reapplication of MOPs [35,37,38]. Sivarajan et al. [38] and Asif et al. [35] in a three-arm compound-design RCT evaluated the effect of multiple intervals of MOPs (every four, eight, or 12 weeks in groups 1, 2, and 3 respectively) on the RTM. The pooled estimate showed that there was no statistically significant difference between the four-week MOPs group (three times of application) and the eight-week MOPs group (two times of application) in the rate of canine retraction in one month (MD = 0.24; 95% CI: -0.28-0.77; p = 0.36, Figure 4). Heterogeneity between the two studies was high (χ2 = 6.57; p = 0.01; I2 = 85%). Also, the MD of 0.06 was found (95% CI: -0.14-0.27; p = 0.55, Figure 5) between the four-week MOPs group and the 12-week MOPs group, which was not statistically significant, with low heterogeneity (χ2 = 1.01; p = 0.31; I2 = 1%). According to GRADE, the overall quality of the evidence supporting this outcome is very low (Table 8).

Jaiswal et al. [37] compared the efficacy of two-time MOPs versus one-time application on the RTM in an SMD RCT. In the first month of canine retraction, no significant differences were found between the two sides of the mouth (x ®= 1.37 ± 0.0.43 mm and x ®= 1.41 ± 0.43 mm, respectively; p = 0.840). On the contrary, the overall canine retraction rate was statistically greater in the two-time MOPs side than the one-time side (x ®= 6.12±0.76 mm and x ®= 4.57 ± 0.54 mm, respectively; p<0.001). According to GRADE, the overall quality of the evidence supporting this outcome is low (Table 8). The difference in the repetition intervals between this study and the other two studies prevented its inclusion in the meta-analysis.

Secondary outcomes

The overall quality of evidence according to GRADE for secondary outcomes is illustrated in Table 8.

Secondary Outcomes Associated With Multiple Methods of Acceleration (Two or More Different Methods of Acceleration)

Two of the included papers in this hub [16,36] evaluated secondary outcomes. Farid et al. [36] assessed molar anchorage loss between the groups of corticotomy + LLLT and corticotomy only. The MD of anchorage loss was greater in the corticotomy only side than the corticotomy + LLLT side (MD: 0.46 ± 2.81 mm). However, this difference was statistically insignificant. Yousif et al. [16] evaluated pain and discomfort between the combined multiple osteoperforation + corticotomy side against the multiple osteoperforation side. No pain and discomfort were reported in both groups. Yousif et al. [16] also assessed canine angulation and inclination. They found that the mean canine angulation changes were greater in the multiple osteoperforation + corticotomy side (x ®= 15.2° ± 1.65°) compared to the multiple osteoperforation side (x ®= 11.0° ± 2.36°) with a statistically significant difference (p = 0.001). Moreover, the distal inclination of the canine was also greater in the multiple osteoperforation + corticotomy side compared to the multiple osteoperforation side with a statistically significant difference (p = 0.001). We could not pool the results of the secondary outcomes to quantitative synthesis due to the use of a different outcome variable between trials.

Secondary Outcomes Associated With Repetition of an Acceleration Method

Two studies in this hub [37,38] discussed the associated secondary outcomes. Sivarajan et al. [38] evaluated the pain and its impact on daily function between the multiple intervals of MOPs (every four, eight, and 12 weeks). Pain associated with MOP was reported in all groups. However, 60% of the MOP-4 patients' group reported moderate pain, with the highest mean pain score (x ®= 1.75 ± 0.72). Moreover, 70% of the MOP-8 patients' group and 75% of the MOP-12 patients' group reported only mild pain with a similar mean pain score (x ®= 1.35 ± 0.59 and x ®= 1.30 ± 0.57, respectively). The impact of this reported pain on daily function centered on speech and chewing; without any statistically significant effect on general activities, like mood and social interaction (p>0.05). Jaiswal et al. [37] estimated molar anchorage loss as well as canine angulation between one-time MOP and two-time MOP sides. Regarding the molar anchorage loss, a statistically insignificant difference (p = 0.657) was found between the one-time MOP side (0.31 ± 0.24 mm) and the side of the two-time MOP (0.30 ± 0.39 mm). Moreover, the canine tipping also demonstrated a statistically insignificant difference in the overall canine retraction intervals (p>0.05), which was 2° in the one-time MOP side and 3° in the two-time MOP. We could not pool the results of the secondary outcomes to quantitative synthesis due to the use of a different outcome variable between trials.

The Strength of the Evidence According to the GRADE Guidelines for the Included Trials

As per the GRADE recommendations, the evidence strength regarding the rate of orthodontics tooth movement as well as the adverse effects ranged from very low to low, as shown in Table 8. The decline in the evidence strength happened because of the risk of bias [16,34-38], indirectness [16,34,35,38], and imprecision [16,34-38].

Discussion

Acceleration of OTM has become a trend in the orthodontic field in recent decades. In the beginning, the trend was to assess the effectiveness of the methods used for OTM acceleration. Subsequently, some clinical trials have been conducted to evaluate the effectiveness of combining two methods or more or repeating a specific method to accelerate the OTM. This approach has not been widely accepted among orthodontic practitioners, and the effectiveness of this combination or repetition is still not known with any certainty.

First theme: multiple methods of acceleration

Unfortunately, the results of the two studies [34,36], which focused on the combination of LLLT with surgical intervention versus a single application of one of them, were contradictory. Abdelhameed and Refai [34] concluded that a combination of MOPs + LLLT achieved a synergistic effect with superior accelerated tooth movement compared to the sole application of each technique. On the contrary, Farid et al. [36] found that the combination of corticotomy + LLLT was not more efficient than the single application of corticotomy only in accelerating canine retraction. The dissimilarity in the findings could be attributed to the fact that the interventions were applied using different protocols. Abdelhameed and Refai [34] applied LLLT from the buccal and palatal surfaces along the root of the canines in addition to the application of MOPs six times over a period of three months, whereas in Farid et al.'s trial [36], the LLLT was applied at the middle point of the canine root on buccal and palatal surfaces, and the corticotomy procedure was performed only once. As mentioned before, the high heterogeneity between the previous studies [34, 6] did not allow for quantitative synthesis of the findings.

However, Yousif et al. [16], who compared the combination of two different surgical interventions against a single application of one of them, reported that the combination outperformed the single surgical technique group as the mean TTM was less in the combination group (67.7 ± 3.09 days) compared to the single-procedure group (85.1 ± 3.03 days). One possible explanation could be the synergistic effect of two surgical techniques with an increased RAP, i.e., increased cortical bone porosity with increased osteoclastic activity following the surgical healing of the cortical bone [39]. On the other hand, the combination of two surgical interventions (corticotomy + MOPs) reduced the treatment duration by 42.8%. This is superior to the acceleration of canine retraction with the MOPs only, which reduced the treatment duration by 25.4% when compared to conventional retraction. This is somewhat consistent with a previous trial, which suggested a possible reduction in the treatment duration of up to 30% [38]. Although surgical methods have been proven to be effective in accelerating OTM [24], different acceleration rates have been published in the literature. A study evaluating traditional corticotomy (with flap elevation) reported that canine retraction increased by two to four times when compared to the non-accelerated group [5], whereas a laser-assisted flapless corticotomy accelerated the canine retraction by 2.5 times when compared to the conventional retraction [40]. On the other hand, flapless piezocision-based corticotomy accelerated canine retraction by 1.5-2 times when compared to the non-accelerated group [2], whereas the application of MOPs produced OTM that was 2.3 times faster than the conventional retraction [41].

Second theme: repetition of an acceleration method

The results of the meta-analysis indicated that the rate of OTM was almost the same after repeated application of MOPs by three times (every four weeks) and two times (every eight or 12 weeks), as shown in Figures 4, 5. However, Jaiswal et al. [37] found the RTM was statistically greater in the two-time MOP side when compared with the one-time MOP side. The previous results could be explained by understanding the mechanism of RAP and the purpose of repeating the acceleration procedures. RAP is a transient phenomenon, beginning a few days after surgery, reaching its highest point at one to two months, and then declining over time [42]. Here is where the role of the repetition of intervention to ensure a continuous activation of the RAP biological response becomes important [43]. Since the purpose of the repetition is the same, which is to re-evoke the RAP and maintain the accelerated OTM, this could be the reason why there is no difference between repetition by two or three times.

The associated secondary outcomes

Considering the associated side effects, the anchorage loss was assessed in two trials, which concluded the same result. Farid et al. [36] found a statistically insignificant difference between the single acceleration method side (corticotomy) and the combined methods side (LLLT + corticotomy). Additionally, Jaiswal et al. [37] demonstrated that anchorage loss did not differ statistically between the sides of one-time MOP and two-time MOP. This could be attributed to the fact that the accelerating interventions were applied topically, leading to localized alveolar response without affecting the posterior segments, and hence anchorage loss did not differ between the two sides of the intervention.

Canine angulation was investigated in two studies. Yousif et al. [16] reported that more distal tipping and more buccal inclination of the canine were shown in the combined acceleration method side (multiple osteoperforation + corticotomy) than the single-method side (multiple osteoperforation). This result can be explained by understanding the biomechanical mechanism. It is known that in sliding techniques, an initial crown tipping is induced first, followed by root uprighting later [44]. The faster the OTM is done, the more tipping of the crown will occur. On the contrary, Jaiswal et al. [37] indicated that the canine tipping was minimal, with non-significant differences between the two sides of one-time MOP and two-time MOP. This can be attributed to the use of a rigid 0.019 x 0.025 stainless steel wire with a closed-coil spring for canine retraction. It is known that rectangular archwire provides good control for tipping during canine sliding; however, the looseness of the archwire fits in the bracket slot and causes a certain degree of tipping [45,46]. Although no important side effects were found in the included three studies, data on periodontal complications, postoperative infection, root resorption, tooth vitality loss, and possible morbidity are not available in the retrieved studies and further research is required.

Limitations of the current review

Only six RCTs (three studies in each theme), which fulfilled the eligibility criteria, were found and included in this SR; four of them were assessed to have some concern of bias, whereas the other two trials were at high risk of bias. The strength of evidence ranged from very low to low. Therefore, there is a need for high-quality RCTs to assess the value of multiplicity or repetition in orthodontic acceleration. Canine retraction following premolar extraction was carried out in all included studies. We could not find trials evaluating acceleration in different treatment strategies such as decrowding of upper and lower teeth, incisors’ retraction or intrusion, and en-masse retraction of anterior teeth. The high heterogeneity and the use of different outcome measures as well as treatment protocols prevented the inclusion of all of the retrieved studies in a meta-analysis and the results of only two studies were pooled. Not all included studies evaluated the side effects associated with the acceleration techniques. Moreover, the studied side effects reported were limited. Therefore, it was difficult to arrive at clear conclusions regarding the accompanying negative effects.

## Conclusions

The combination of LLLT with a surgical technique seemed to outperform the application of each technique separately when accelerating tooth movement. The combination of two surgical interventions may produce a synergistic effect leading to a reduced treatment time compared to the application of a single surgical intervention. The quality of evidence of previous conclusions was very low. The double application of surgical procedures appears to be more effective than the single application but the quality of evidence in this aspect is low. As the strength of evidence of the reported results ranged from low to very low, we confirm the need for more well‑conducted RCTs evaluating the benefits of combining several acceleration techniques throughout the treatment procedure or repeating specific methods in comparison with the single application. Future research should also consider the broad spectrum of possible side effects accompanying multiple or repeated applications.
